# Microbial Production of Bioactive Retinoic Acid Using Metabolically Engineered *Escherichia coli*

**DOI:** 10.3390/microorganisms9071520

**Published:** 2021-07-16

**Authors:** Minjae Han, Pyung Cheon Lee

**Affiliations:** Department of Molecular Science and Technology, Ajou University, World Cup-ro, Yeongtong-gu, Suwon-si 16499, Gyeonggi-do, Korea; oneminzaeh@ajou.ac.kr

**Keywords:** retinoid, retinoic acid, metabolic engineering, retinaldehyde dehydrogenase, β-carotene 15,15′-oxygenase

## Abstract

Microbial production of bioactive retinoids, including retinol and retinyl esters, has been successfully reported. Previously, there are no reports on the microbial biosynthesis of retinoic acid. Two genes (*blh_SR_* and *raldh_HS_*) encoding retinoic acid biosynthesis enzymes [β-carotene 15,15′-oxygenase (Blh) and retinaldehyde dehydrogenase2 (RALDH2)] were synthetically redesigned for modular expression. Co-expression of the *blh_SR_* and *raldh_HS_* genes on the plasmid system in an engineered β-carotene-producing *Escherichia coli* strain produced 0.59 ± 0.06 mg/L of retinoic acid after flask cultivation. Deletion of the *ybbO* gene encoding a promiscuous aldehyde reductase induced a 2.4-fold increase in retinoic acid production to 1.43 ± 0.06 mg/L. Engineering of the 5’-UTR sequence of the *blh_SR_* and *raldh_HS_* genes enhanced retinoic acid production to 3.46 ± 0.16 mg/L. A batch culture operated at 37 °C, pH 7.0, and 50% DO produced up to 8.20 ± 0.05 mg/L retinoic acid in a bioreactor. As the construction and culture of retinoic acid–producing bacterial strains are still at an early stage in the development, further optimization of the expression level of the retinoic acid pathway genes, protein engineering of Blh and RALDH2, and culture optimization should synergistically increase the current titer of retinoic acid in *E. coli*.

## 1. Introduction

Retinoids (or vitamin A and its analogs) are essential components of visual function, cell differentiation, and other cellular signaling pathways [1,2]. Retinoids are lipophilic compounds with diverse structures, based on their end groups. They are composed of three structural moieties: a β-ionone ring, an isoprenoid backbone, and a functional group such as an alcohol (retinol), an aldehyde (retinal), a carboxylic acid (retinoic acid), or an ester group (retinyl esters) [3]. In a biotechnological aspect, retinoids are used as a dermatological agent against acne, psoriasis, skin aging, and other skin conditions [4].

**Figure 1:**
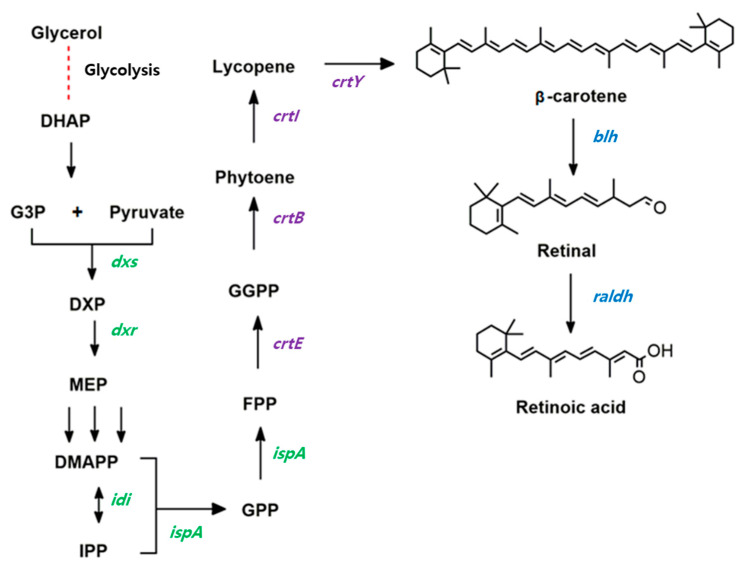
Redesigned biosynthetic pathway of retinoic acid from β-carotene in recombinant *Escherichia coli*. The genes involved in this redesigned pathway are *dxs* (encoding1-deoxy-d-xylulose-5-phosphate synthase); *dxr* (1-deoxy-d-xylulose-5-phosphate reductoisomerase); *idi* (isopentenyl diphosphate isomerase); *ispA* (farnesyl diphosphate synthase); *crtE* (geranylgeranyl diphosphate synthase); *crtB* (phytoene synthase); *crtI* (phytoene desaturase); *crtY* (lycopene cyclase); *blh* (β-carotene 15,15′-oxygenase); and *raldh* (retinal dehydrogenase).

Retinoic acid is one of the most important ingredients in cosmetic skincare products because it can protect against UV-radiation-induced skin damage in fibroblasts and other skin cells [5]. Therefore, there is a high demand for retinoic acid in the cosmetic and pharmaceutical industries [6]. In a biological system, retinoic acid is biosynthesized from β-carotene in two reaction steps (Figure 1). β-carotene is symmetrically cleaved by β-carotene 15,15′-oxygenase (Blh), generating retinaldehyde (also known as retinal), which is then oxidized to retinoic acid by retinal dehydrogenase (Raldh). The retinoic acid precursor β-carotene is synthesized by four biosynthetic pathway enzymes [7], known as geranylgeranyl diphosphate synthase (CrtE), phytoene synthase (CrtB), phytoene desaturase (CrtI), and lycopene cyclase (CrtY) (Figure 1).

Most retinoic acids are commercially produced via chemical synthesis [8]. Microbial production of RA has received attention as an alternative process for retinoic acid. Although retinol and retinyl ester have been produced in microbial host strains, including *Escherichia coli* [9,10,11] and *Saccharomyces cerevisiae* [12], there are no reports on the microbial biosynthesis of retinoic acid. As *E. coli* has been exploited as a microbial host system for retinoid production, it can serve as a host for the redesign and reconstruction of the retinoic acid pathway. 

In this study, the retinoic acid biosynthesis pathway was reconstructed by co-expressing Blh from *Salinibacter ruber* and Raldh from the Hep3B cell line in a metabolically engineered β-carotene-producing *E. coli*. Next, retinoic acid titer was enhanced by deleting a gene encoding a promiscuous enzyme which reduced retinoic acid titer, engineering of the 5’-UTR sequence of the two retinoic acid pathway genes, and optimization of the culture conditions of a retinoic acid–producing strain in a bioreactor.

## 2. Materials and Methods

### 2.1. Strains, Media, and Culture Conditions

All *E. coli* strains and plasmids used in this study are listed in Table 1. The *E. coli* Top 10 strain was used for gene cloning, and *E. coli* BL21(DE3) was used for protein expression. The *E. coli* strains were grown in Luria-Bertani (LB) broth (10 g/L tryptone, 5 g/L yeast extract, and 5 g/L NaCl) at 37 °C with shaking at 250 rpm. For retinoid production, the *E. coli* MG1655 strain was grown in Terrific Broth (TB) medium (12 g/L of tryptone, 24 g/L of yeast extract, 0.17 M KH_2_PO_4_, and 0.72 M K_2_HPO_4_) supplemented with 20 g/L of glycerol on a rotary shaker at 30 °C and 250 rpm. The Hep3B cell line was a gift from Professor Wook Kim (Ajou University, Suwon, Korea).

### 2.2. Plasmid Construction for the Expression of Retinoid Biosynthesis Pathway Enzymes 

The *blh_SR_* gene encoding β-carotene 15,15′-oxygenase (BlhSR) from *S. ruber*, which was previously cloned in the plasmid pUCMr-blh [11], was utilized, and the *raldh_HS_* gene encoding retinaldehyde dehydrogenase 2 (RALDH2, GenBank accession number, AB015226.1) from the Hep3B cell line was cloned into pUCMr to construct pUCMr-raldh (Table 1). To construct the retinoic acid pathway, the two genes encoding BlhSR and RALDH2 were redesigned as an individual expression module, and then assembled into the plasmid pSTVM2, using the USER^®^ cloning method, resulting in pSTVM2-blh-raldh.

To enhance the expression levels of the two genes in *E. coli*, the 5′-untranslated regions (5′-UTRs) of the two genes were engineered by using 26-bp mRNA-stabilizing sequences, which were obtained by using UTR designer (http://sbi.postech.ac.kr/utr_designer, accessed on 7 May 2020). Three 5′-UTRs of blhSR (UTR12, UTR37, and UTR46), which were investigated in our previous study [11], were utilized as pUCMr12-blh, pUCMr37-blh, and pUCMr46-blh, respectively, in this study. In a similar way described in [11], two 5′-UTRs of RALDH2 (named UTRH for high expression, and UTRM for moderate expression) were predicted using UTR designer, and two plasmids (pUCMrH-raldh and pUCMrM-raldh) were constructed. Next, the two genes with corresponding 5′-UTR sequences were subcloned into pSTVM2, using Gibson assembly [3], resulting in pSTVM2-12blh-raldh, pSTVM2-37blh-raldh, pSTVM2-46blh-raldh, pSTVM2-blh-Hraldh, pSTVM2-blh-Mraldh, pSTVM2-37blh-Hraldh, and pSTVM2-37blh-Mraldh (Table 1).

### 2.3. Deletion of the ybbO Gene in the BETA-1 Strain

The *ybbO* gene, which encodes a putative oxidoreductase in the BETA-1 strain, was deleted through one-step homologous recombination, using the pRed/ET-mediated recombination method (Gene Bridges, Heidelberg, Germany). A *ybbO*-deleting linear DNA fragment consisting of a 50-bp left homology arm sequence, a FRT-Km^r^-cassette, and a 50-bp right homology arm sequence was constructed by using the USER cloning kit (New England Biobabs, Ipswich, MA, USA) with specific primers (Supplementary Materials Table S1). The *ybbO*-deletion mutants were selected on LB agar plates containing 30 µg/mL kanamycin, followed by the generation of a Km^R^ marker-free strain, using an FLP recombinase, which was inducibly expressed in the temperature-sensitive pCP20 helper plasmid. The sequence of the deletion site in the BETA-1 strain was verified via Sanger sequencing of the isolated gDNA. The resultant *ybbO*-deletion strain was named BETA-1∆ybbO.

### 2.4. Integrating Two Retinoic Acid Pathway Genes into the BETA-1∆ybbO Strain

The BETA-1∆ybbO∆malT::37blh strain expressing UTR37-*blh_SR_* on the genome was constructed by integrating a synthetic module expressing UTR37-*blh_SR_* into a *mal*T site in BETA-1∆ybbO. Similarly, the BETA-1∆ybbO∆malT:: Mraldh strain was created through the integration of a synthetic module expressing UTRM-*raldh* into the *mal*T site in BETA-1∆ybbO. BETA-1∆ybbO∆malT::37blh::Mraldh was constructed by integrating a synthetic module co-expressing UTR37-*blh_SR_* and UTRM-*raldh* into the *mal*T site in BETA-1∆ybbO. The abovementioned genome integrations were performed by using the CRISPR/Cas9 genome-editing system. Linear DNA fragments containing 250-bp homology arm sequences were constructed by using overlapping PCR with gene-specific primers (Supplementary Materials Table S1). The guide RNA (gRNA) sequence was designed by using the CHOPCHOP program (https://chopchop.cbu.uib.no/, accessed on 10 September 2020). The pgRNA_malT vector was constructed via PCR-mediated amplification of the pgRNA plasmid backbone with primers containing 20 bp of the gRNA sequence. Genome-editing strains were selected via colony PCR. The full sequence of the edited site in BETA-1∆ybbO was verified through Sanger sequencing of the isolated gDNA (Macrogen, Seoul, Korea).

### 2.5. Reverse-Transcription PCR (RT-PCR) 

Total RNA was extracted from *E. coli* cells expressing the *blh*_SR_, UTR-12*blh*, UTR-37*blh*, UTR-46*blh*, *raldh*, UTRM-*raldh*, or UTRM-*raldh* genes, which were grown in the mid-exponential growth phase, using an easy-BLUE™ Total RNA Extraction Kit (Intron, Seoul, Korea). For reverse transcription-polymerase chain reaction (RT-PCR) analysis, cDNA was synthesized from the total RNA samples, using the ReverTra™Ace qPCR RT Kit (Toyobo, Osaka, Japan). PCR products were then analyzed on a 1% (*w*/*v*) agarose gel. The RT-PCR conditions were as follows: denaturation at 95 °C for 1 min; 30 cycles of denaturation at 95 °C for 30 s, annealing at 60 °C for 30 s, and extension at 72 °C for 20 s. The *cysG* gene encoding siroheme synthase was used as a reference gene. The primers used for the RT-PCR are listed in Supplementary Materials Table S1. 

### 2.6. SDS-PAGE and Western Blot Analysis

To fuse the 6 × His amino acid sequence into the N-terminus of the BlhSR and RALDH2 proteins, the *blh*_SR_ and *raldh* genes were cloned into the pET21α(+) plasmid to construct pET21-blhSR and pET21-raldh, respectively. *E. coli* BL21 (DE3) harboring pET21α(+), pET21-blhSR, or pET21-raldh was grown to an OD_600_ of 0.6–0.8, then 1 mM of isopropyl β-d-thiogalactopyranoside (IPTG) was added to the culture medium. Three hours after induction, the cells were harvested, washed twice with 50 mM Tris-HCl buffer (pH 6.8), and disrupted via sonication. The crude protein extracts were separated on a 12% (*w*/*v*) SDS-PAGE gel. The gels were subsequently stained with Coomassie Brilliant Blue to visualize the protein bands. For Western blot analysis, the separated protein bands on the gels were transferred to a PVDF membrane, using Trans-Blot SD semi-dry (BioRad, Hercules, MA, USA) for 1 h, at 25 V. The blot was blocked with Tris-buffered saline with Tween20 (TBST) containing 5% (*w*/*v*) skim milk for 2 h and washed three times with TBST, at 25 °C. SuperSinalTM West Pico PLUS Chemiluminescent substrate solution (Thermo Fisher Scientific, Waltham, MA, USA) was added for the immunodetection of 6×His-tagged BlhSR and RALDH2 on the membrane.

### 2.7. Bioreactor Fermentation

Batch fermentation was carried out in a 5 L BioFlo 320 bioreactor (Eppendorf, Hamburg, Germany) containing 1.5 L of TB medium (supplemented with 20 g/L glycerol and 50 ug/mL chloramphenicol) under different conditions: culture temperature (20, 30, and 37 °C), culture pH values (6.0, 6.5, and 7.0) and dissolved oxygen (DO) levels (>10%, 30%, and 50%). The pH was maintained at a preset value through the automatic addition of 24% (*v*/*v*) NH_4_OH and 2 N HCl. The DO level was controlled by increasing the agitation rate from 200 to 600 rpm and by supplying air and pure O_2_ gas. Cell growth was monitored at 600 nm (OD_600_), using a SpectraMax Plus384 spectrophotometer (Molecular Devices, San Jose, CA, USA).

### 2.8. Extraction of Retinoids

Retinoid-producing cells were harvested and extracted with 15 mL of acetone until all visible colors were extracted. To the acetone extract, 15 mL of 5 N NaCl solution was added, and then the pH of the mixed solution was adjusted to 2.0 by adding 85% phosphoric acid (Sigma-Aldrich, Saint Louis, MO, USA). Next, an equal volume of hexane was added to the acidified mixed solution and mixed well. After centrifugation for 5 min at 3800 rpm, the upper solvent layer, which contained the retinoids, was collected and dehydrated over anhydrous sodium sulfate. The solution was then completely dried in a Genevac EZ2 centrifugal evaporator (SP Industries, Warminster, PA, USA). The dried residue was resuspended in 1 mL acetone and stored at −20 °C, in the dark, until analysis. 

### 2.9. Extract Analysis Using HPLC and Mass Spectrometry (MS)

A 5 µL aliquot of the organic extract was injected into an Agilent 1260 high-performance liquid chromatography (HPLC) system (Agilent Technologies, Santa Clara, CA, USA) equipped with a photodiode array detector (Agilent Technologies) and a Poroshell 120 EC-C18 column (2.1 × 50 mm, 2.7 µm; Agilent Technologies). The column temperature was maintained at 23 °C, while the flow rate was maintained at 0.4 mL/min. Two mobile-phase systems were used for gradient elution: mobile phase A (methanol, acetonitrile, and acetic acid, 70.0:30.0:0.1, *v*/*v*) and mobile phase B (acetonitrile, methanol, water, isopropanol, and acetic acid, 60.0:20.0:19.0:5.0:0.1, *v*/*v*). The linear gradient was generated as follows: minutes 0–5, 100% B; minutes 5–6, 100% B to 100% A; minutes 6–28, 100% A; minutes 28–29, 100% A to 100% B; and minutes 29–35, 100% B. The mass fragmentation spectrum of retinoic acid was monitored in positive mode on an LC–MS 6150 quadrupole system (Agilent Technologies) equipped with an atmospheric-pressure chemical ionization interface. The MS conditions used were described in our previous study [11].

## 3. Results

### 3.1. Expression of Retinoic Acid Pathway Enzymes

**Figure 2:**
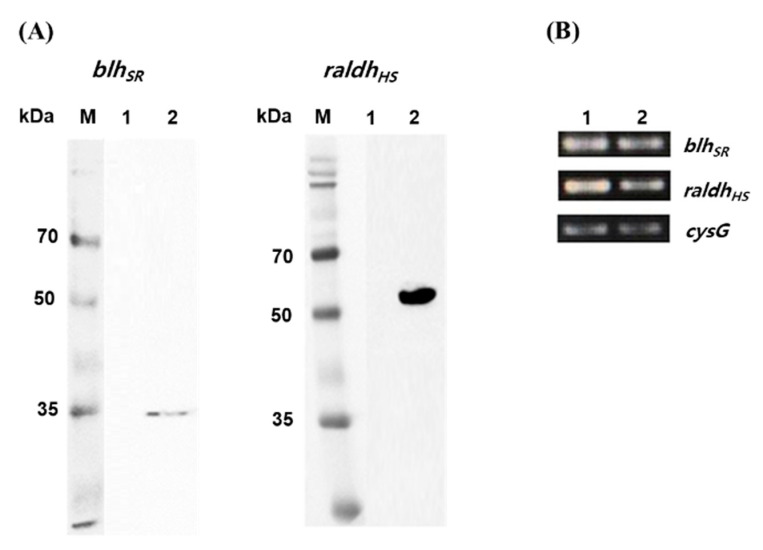
Western blot analysis and mRNA transcription of *blh_SR_* and *raldh_HS_* genes. (**A**) Western blot analysis of the protein expression of *blh_SR_* (**left**) and *raldh_HS_* (**right**) in the *E. coli* BL21 (DE3) strain. Lane 1 in each panel indicates the total extracted proteins from BL21 (DE3)/pET21α(+) while lane 2 indicates the total extracted proteins from BL21 (DE3)/pET21-blhSR (left), and BL21 (DE3)/pET21-raldh (right). (**B**) Total RNA was isolated from the *E. coli* BETA-1 strain in the (1) mid-log and (2) stationary growth phases, and then the transcript levels of *blh_SR_* and *raldh_HS_* were analyzed via RT-PCR. The *cysG* gene was used as the reference gene.

As a precursor-producing strain, the *E. coli* BETA-1 strain (unpublished) was used to construct the retinoic acid pathway. The BETA-1 strain has an engineered expression system for both the isopentenyl diphosphate and β-carotene biosynthetic pathways. Heterologous expression of two key retinoic acid pathway genes (*blh_SR_* from *S. ruber* and *raldh_HS_* from the Hep3B cell line) was investigated in two *E. coli* strains. When the *blh_SR_* and *raldh_HS_* genes were induced through the 6×His tagging system [pET21α(+) plasmid] in *E. coli* BL21 (DE3), one band corresponding to each gene was detected during immunoblotting (Figure 2A). The expected protein size (34.7 kDa of *blh_SR_* and 56.8 kDa of *raldh_HS_*) and one band of each gene indicated that *blh_SR_* and *raldh_HS_* were expressed without protein degradation in the heterologous host *E. coli*. In addition, the mRNA transcription of each gene in the mid-log (at 36 h culture) and stationary growth (at 48 h culture) phases of the BETA-1 strain was confirmed via RT-PCR analysis (Figure 2B).

### 3.2. Construction of Retinoic Acid Biosynthetic Pathway in the E. coli BETA-1 Strain 

**Figure 3:**
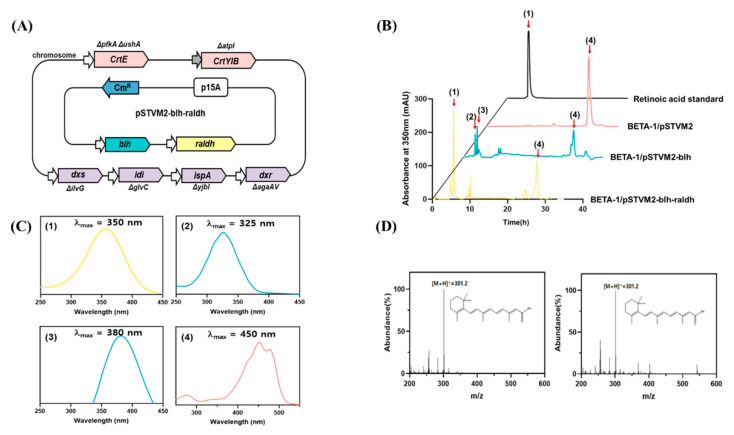
Reconstruction of the retinoic acid pathway and retinoid profiles in *E. coli*. (**A**) Schematic description of the metabolically engineered BETA-1 strain harboring the pSTVM2-blh-raldh plasmid expressing the Blh and ALDH1A2 enzymes for the biosynthesis of retinoic acid. (**B**) HPLC analysis of commercial standard retinoic acid and retinoid extracts from three recombinant *E. coli* strains. Peak 1 corresponds to retinoic acid; peak 2, retinol; peak 3, retinal; peak 4, β-carotene. (**C**) UV/Vis spectrum analysis of compounds corresponding to peaks 1–4 in the HPLC chromatogram. (**D**) LC–mass spectrometry analysis of a retinoic acid standard (left) and retinoic acid extracted from *E. coli* (right).

To reconstruct the retinoic acid pathway in the BETA-1 strain, the *blh_SR_* and *raldh_HS_* genes were made modular and assembled into the plasmid pSTVM2, constructing pSTVM2-blh-radlh (Table 1). After the strain BETA-1/pSTVM2-blh-radlh (Figure 3A) was constructed, the acetone extract of the BETA-1/pSTVM2-blh-radlh grown in 100 mL flasks was analyzed together with the extracts of two control strains (BETA-1/pSTVM2 and BETA-1/pSTVM2-blh), using HPLC. One new peak (peak 1 in Figure 3B) in the extract of BETA-1/pSTVM2-blh-radlh was detected at the same retention time as that of the retinoic acid standard, within the same UV/Vis spectrum (Figure 3C). LC–MS analysis revealed that peak 1 in the extract of the BETA-1/pSTVM2-blh-radlh extract corresponded to a similar molecular fragment pattern and the same parent ion of *m*/*z* 301.2 [M + H]^+^ as that of a retinoic acid standard (Figure 3D). Collectively, the *blh_SR_* and *raldh_HS_* genes were functionally expressed in the BETA-1/pSTVM-blh-raldh strain, generating retinoic acid (0.52 ± 0.10 mg/L) from β-carotene. Notably, the presence of retinol (peak 2) and retinal (peak 3) in the extract of BETA-1/pSTVM2-blh was the same as that in our previous study [11].

### 3.3. Enhanced Retinoic Acid Production through Deletion of the ybbO Gene in the BETA-1 Strain

**Figure 4:**
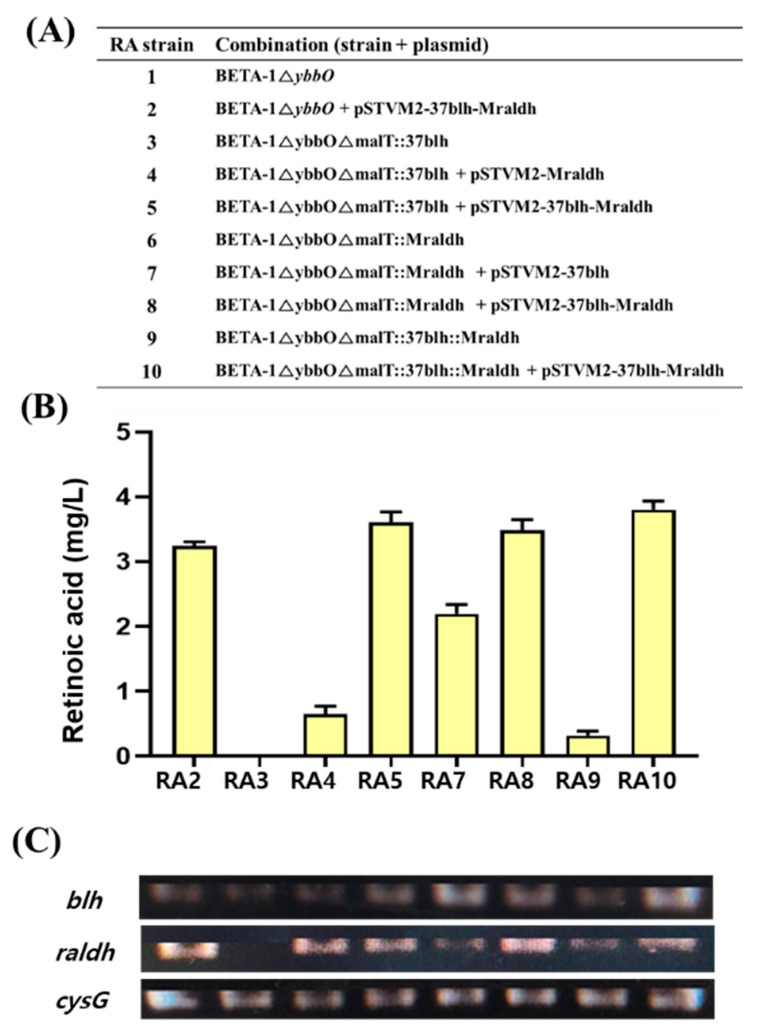
Retinoic acid production and mRNA expression level of the UTR37-*blh_SR_* and UTRM-*raldh* genes in retinoic acid–producing strains. (**A**) List of strains constructed through genome editing and transforming plasmids into the genome-edited strains. (**B**) Retinoic acid production from eight metabolically engineered strains grown in flask cultures. (**C**) mRNA expression level of UTR37-*blh_SR_* and UTRM-*raldh* genes in ten strains grown in flask cultures. The *cysG* gene, encoding siroheme synthase, was used as a reference gene.

It has been reported that endogenous aldehyde reductases, such as *ybbO* in *E. coli*, showed unexpected activity similar to retinol reductase, an enzyme that converts retinal to retinol [9]. Therefore, the production of retinoic acid in BETA-1/pSTVM2-blh-radlh might be enhanced by limiting the conversion of retinal to retinol by suppressing endogenous aldehyde reductase activity. As a proof of this hypothesis, the *ybbO* gene was deleted in the genome of the BETA-1 strain, creating the BETA-1ΔybbO strain (named as RA1, Figure 4A). Quantitative analysis of acetone extracts of the strain RA1/pSTVM2-blh-radlh and the control strain BETA-1/pSTVM2-blh-radlh revealed that the RA1/pSTVM2-blh-radlh strain produced 1.43 ± 0.06 mg/L of retinoic acid, which was 2.4 times higher than what the BETA-1/pSTVM2-blh-radlh strain produced (0.59 ± 0.06 mg/L). No presence of retinal and retinol was observed in both strains, indicating that retinal was completely converted into retinoic acid. The increased titer of retinoic acid in the RA1/pSTVM2-blh-radlh strain can be explained by the fact that more retinal could be transformed into retinoic acid (Figure 1) in that strain. This is supported by the experiment of the two *blh*-expressing strains, where higher retinal (0.47 ± 0.1 mg/L) and lower retinol (0.25 ± 0.05 mg/L) titers were measured in the RA1/pSTVM2-blh strain in comparison with titers of retinal (0.14 ± 0.06 mg/L) and retinol (0.37 ± 0.06 mg/L) in the control BETA-1/pSTVM2-blh strain.

### 3.4. Transcription Control Engineering of blh_SR_ and raldh_HS_ Genes for Improving Retinoic Acid Production

As a strategy for enhancing retinoic acid production, the mRNA-stabilizing region (mRS) engineering approach [11] was adopted to increase the expression of *blh_SR_* and *raldh_HS_*. Three previously studied synthetic sequences (UTR12, UTR37, and UTR46) [11] for regulating the expression of the *blh_SR_* gene were assembled with *raldh_HS_* into pSTVM2, constructing three retinoic acid–producing synthetic mRS-blh-raldh expression vectors: pSTVM2-12blh-raldh, pSTVM2-37blh-raldh, and pSTVM2-46blh-raldh. Quantitative analysis of the four strains (RA1/pSTVM2-12blh-raldh, RA1/pSTVM2-37blh-raldh, RA1/pSTVM2-46blh-raldh, and RA1/pSTVM2-blh-raldh as a control) revealed that the RA1/pSTVM2-37blh-raldh strain produced the highest amount of retinoic acid (2.63 ± 0.14 mg/L) among them. Similar to the design of mRS for *blh_SR_*, two mRS synthetic sequences (UTRH and UTRM for high and moderate expression, respectively) for *raldh_HS_*, were designed and assembled with UTR37-blh or blh into pSTVM2, resulting in two synthetic mRS-blh-mRS-raldh expression vectors (pSTVM2-37blh-Hraldh and pSTVM2-37blh-Mraldh) and two synthetic blh-mRS-raldh expression vectors (pSTVM2-blh-Hraldh and pSTVM2-blh-Mraldh). Quantitative analysis of the four strains (RA1/pSTVM2-37blh-Hraldh, RA1/pSTVM2-37blh-Mraldh, pSTVM2-blh-Hraldh, and pSTVM2-blh-Mraldh) revealed that the RA1/pSTVM2-37blh-Mraldh strain produced the highest amount of retinoic acid (3.46 ± 0.16 mg/L), which was 2.4 times higher than 1.43 ± 0.07 mg/L in the non-mRS-engineered RA1/pSTVM2-blh-raldh, 2.0 times higher than 1.71 ± 0.10 g/L in the mRS-raldh engineered RA1/pSTVM2-blh-Mraldh, and 1.3 times higher than 2.63 ± 0.14 mg/L in the mRS-blh engineered RA1/pSTVM2-37blh-raldh.

### 3.5. Construction and Expression of Retinoic Acid Biosynthetic Pathway Genes on the Genome of RA1 Strain

To integrate the retinoic acid pathway genes in the RA1 strain, two synthetic modules expressing each individual gene in UTR37-*blh* and UTRM-*raldh* and a synthetic module co-expressing two genes in UTR37-*blh* and UTRM-*raldh* were integrated into the *malT* site of the RA1 strain, using the CRISPR/Cas9 system (Figure 4A). The three genome-edited strains were BETA-1∆ybbO∆malT::37blh (RA3), BETA-1∆ybbO∆malT::Mraldh (RA6), and BETA-1∆ybbO∆malT::37blh::Mraldh (RA9). To compare retinoic acid production in the three strains, the genome-edited RA3 strain was transformed with pSTVM2-Mraldh and pSTVM2-37blh-Mraldh, resulting in the RA3/pSTVM2-Mraldh (RA4) and RA3/pSTVM2-37blh-Mraldh (RA5) strains, respectively. Similarly, two strains, RA7 (RA6/pSTVM2-37blh) and RA8 (RA6/pSTVM2-37blh-Mraldh), were constructed from the genome-edited RA6 strain, and the RA10 (RA9/pSTVM2-37blh-Mraldh) strain was created from the genome-edited RA9 strain by transforming with the corresponding plasmids. When the retinoic acid titer was compared between the RA2 strain, where UTR37-*blh* and UTRM-*raldh* were expressed on a multi-copy plasmid system, and the RA9 strain, where UTR37-*blh* and UTRM-*raldh* were expressed on the genome system, the retinoic acid titer (0.32 ± 0.05 mg/L) of RA9 strain was approximately 7 times less than 3.24 ± 0.05 mg/L of retinoic acid in the RA2 strain (Figure 4B). The difference of retinoic acid titer in the RA2 and RA9 strains may be due to the different expression levels of UTR37-*blh* and UTRM-*raldh* genes in RA2 and RA9. The mRNA levels of UTR37-*blh* and UTRM-*raldh* in RA2 were about two-fold higher than that in RA9 (Figure 4C). The highest amount of retinoic acid at 3.80 ± 0.11 mg/L was produced in the RA10 strain grown in flask culture. 

### 3.6. Bioreactor Study of Retinoic Acid–Producing Strains

**Figure 5:**
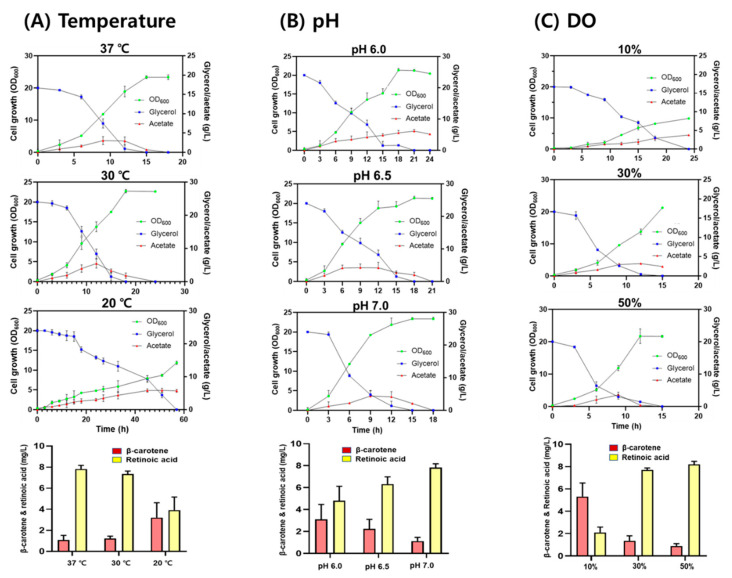
Batch fermentation of the RA10 strain under different culture conditions. (**A**) Effect of temperature (20, 30, and 37 °C) on cell growth at pH 7.0 and a DO of 30%. (**B**) Effect of pH (6.0, 6.5, and 7.0) on cell growth at 37 °C and a DO of 30%, (**C**) Effect of DO (10%, 30%, and 50%) on cell growth at pH 7.0 and 37 °C. Cell growth (OD_600_) is represented as a green circle; glycerol concentration, a blue square; acetate concentration, a red triangle.

To investigate the effect of culture conditions on retinoic acid production, the RA10 strain was grown in a bioreactor containing TB medium under different culture conditions (temperature, pH, and DO levels). When the RA10 strain was grown at temperatures of 20, 30, and 37 °C, at pH 7.0, and a DO of 30%, retinoic acid production was 3.92 ± 1.25 mg/L, 7.35 ± 0.28 mg/L, and 7.82 ± 0.35 mg/L, respectively. The final cell concentration at both 30 and 37 °C was similar but decreased by approximately 45% at 20 °C (Figure 5A). When the RA10 strain was grown at pH values of 6.0, 6.5, and 7.0 at 37 °C and a DO of 30%, retinoic acid production was 4.80 ± 1.32 mg/L, 6.30 ± 0.68 mg/L, and 7.82 ± 0.35 mg/L, respectively. The final cell concentrations were similar at all pH values (Figure 5B). When the RA10 strain was grown at a DO of 10%, 30%, and 50% at pH 7.0 and 37 °C, retinoic acid production was 2.10 ± 0.49 mg/L, 7.70 ± 0.18 mg/L, and 8.20 ± 0.05 mg/L, respectively. The final cell concentrations at both 50% and 30% DO were similar, but significantly reduced to 25% at 10% DO (Figure 5C). The decreased cell growth at 10% DO may be due to the high accumulation of acetic acid in the medium caused by the activated fermentative metabolism, triggering lower oxygen availability. Under all tested conditions, 20 g/L glycerol was completely consumed, and acetic acid was produced up to 5.10 ± 0.21 g/L (Figure 5B,C). Based on a retinoic acid titer (mg-retinoic acid/L) and a conversion yield (mg-retinoic acid/g-glycerol), 37 °C, pH 7.0, and 50% DO were optimum conditions to produce retinoic acid in the RA10 strain.

## 4. Discussion

The production of retinoids, including retinol and retinyl ester, from microbes has been successfully reported. To date, there are no reports on the microbial biosynthesis of retinoic acid. Recently, one study reported that retinoic acid could be produced from retinol in an in vitro system utilizing a bacterial aldehyde dehydrogenase [14]. Therefore, to construct a platform microbial strain for retinoic acid production, two genes (*blh_SR_* and *raldh_HS_*) encoding retinoic acid biosynthesis pathway enzymes, β-carotene 15,15′-oxygenase (Blh) [11] and retinaldehyde dehydrogenase 2 (RALDH2), were synthetically redesigned to be modularly expressed [15]. Co-expression of the *blh_SR_* and *raldh_HS_* genes on the plasmid system in the BETA-1 strain produced 0.59 ± 0.06 mg/L of retinoic acid in flask cultivation (Figure 3). Notably, it has been thought that mammalian RALDHs did not show retinal activity in microbial hosts [14]. However, the *raldh_HS_* gene encoding mammalian RALDH2 from the Hep3B cell line was functionally transcribed (analyzed via RT-PCR) and translated (analyzed through Western blot) in a microbial host, *E. coli* (Figure 2). Retinoic acid was produced from retinal that was generated from β-carotene by Blh in the BETA-1 strain.

To increase the retinoic acid titer, three approaches were used: (1) deletion of the *ybbO* gene encoding a promiscuous aldehyde reductase, (2) engineering of the 5′-UTR sequence of the *blh_SR_* and *raldh_HS_* genes, and (3) optimization of culture conditions of retinoic acid–producing strains in a bioreactor. A 2.4-fold enhancement in retinoic acid production, from 0.59 ± 0.06 mg/L to 1.43 ± 0.06 mg/L, was observed after the deletion of the *ybbO* gene, which decreased retinoic acid titer through the reduction of retinal to retinol. The gene deletion effect was confirmed by observing a higher production (0.47 ± 0.1 mg/L) of the retinoic acid precursor retinal in the BETA-1ΔybbO strain expressing only Blh than that (0.14 ± 0.06 mg/L) of the BETA-1 strain expressing only Blh. Therefore, limiting the activity of promiscuous aldehyde reductases is crucial for the production of retinoic acid in heterologous microbial strains. Engineering of the 5′-UTR sequence to increase the expression level of pathway enzymes, especially in heterologous hosts, has been successfully exploited in *E. coli* [11,16,17] and yeasts [18]. Further, 5′-UTR engineering was successfully applied to the *blh_SR_* and *raldh_HS_* genes of the retinoic acid biosynthesis pathway, consequently increasing the retinoic acid yield from 1.43 ± 0.06 mg/L to 3.46 ± 0.16 mg/L in flask cultivation. Finally, optimized culture conditions (37 °C, pH 7.0, and 50% DO) enhanced retinoic acid production of up to 8.20 ± 0.05 mg/L in a bioreactor (Figure 5).

Even though the retinoic acid pathway genes were co-expressed on the genome of the BETA-1ΔybbO strain, the retinoic acid titer was 10 times less (0.32 ± 0.05 mg/L) than that obtained via co-expression on a plasmid in the BETA-1ΔybbO strain (Figure 4). This suggests that the optimization of the expression levels of retinoic acid pathway genes is crucial, and thus should be regarded as a target for the increased production of retinoic acid. In addition, as a precursor, β-carotene accumulated without being converted into retinal, so the protein engineering of Blh is needed to have higher activity on β-carotene. Finally, as the construction and culture of retinoic acid–producing strains is at an early stage in the development of the microbial processes for retinoic acid production, further optimization of the fermentation operations (such as fed-batch cultivation), media, and intensive strain improvement should increase the current titer of retinoic acid in *E. coli*.

## Figures and Tables

**Table 1:** Strains and plasmids used in this study.

Strains and Plasmids	Relevant Properties	Source or Reference
Strains		
*E. coli* TOP10	F- mcrA Δφ80lacZΔM15 ΔlacX74 nupG recA1 araD139 Δ(ara-leu)7697 galE15 galK16 rpsL(Str^R^) endA1	Invitrogen
*E. coli* BL21(DE3)	F^–^*omp*T*galdcmlonhsd*S_B_(*r_B_*^–^*m_B_*^–^) λ(DE3 [*lac*I*lac*UV*5*-T*7p07 ind*1 *sam*7 *nin*5]) [*mal*B^+^]_K-12_(λ^S^)	NEB
BETA-1	B120 ::idi ::ispA :: dxs :: dxr :: CrtEX2 :: *P*trc_YIB_PAG (atpI site)	unpublished
BETA-1∆ybbO	Deletion of *ybbO* in BETA-1	This study
BETA-1∆ybbO∆malT::37blh	Integration of UTR37-blh in BETA-1∆ybbO	This study
BETA-1∆ybbO∆malT:: Mraldh	Integration of UTRM-raldh in BETA-1∆ybbO	This study
BETA-1∆ybbO∆malT::37blh::Mraldh	Integration of UTR37-blh and UTRM-raldh in BETA-1∆ybbO	This study
Plasmids for pathway construction		
pUCM	Cloning vector modified from pUC19; constitutive lac promoter, Ap	
pUCMr	Cloning vector modified from pUCM; constitutive lac promoter and rop gene, Ap (low copy plasmid)	[11]
pSTVM2	Cloning vector modified from pSTV29; constitutive *lac* promoter, Cm	
pUCMr-blh	Constitutive expressed *blh* gene from *S. ruber*	[11]
pUCMr12-blh	Constitutive expressed *blh* gene from *S. ruber* with UTR12 sequence	[11]
pUCMr37-blh	Constitutive expressed *blh* gene from *S. ruber* with UTR37 sequence	[11]
pUCMr46-blh	Constitutive expressed *blh* gene from *S. ruber* with UTR46 sequence	[11]
pUCMr-raldh	Constitutive expressed *raldh* gene from *Hep3B cell line*	This study
pUCMrH-raldh	Constitutive expressed *raldh* gene from *Hep3B cell line* with UTRH sequence	This study
pUCMrM-raldh	Constitutive expressed *raldh* gene from *Hep3B cell line* with UTRM sequence	This study
pSTVM2-blh-raldh	Constitutive expressed *blh* and *raldh* genes	This study
pSTVM2-37blh	Constitutive expressed UTR37-*blh* gene	This study
pSTVM2-Mraldh	Constitutive expressed UTRM- *raldh* gene	This study
pSTVM2-12blh-raldh	Constitutive expressed UTR12-*blh* gene and *raldh* gene	This study
pSTVM2-37blh-raldh	Constitutive expressed UTR37-*blh* gene and *raldh* gene	This study
pSTVM2-46blh-raldh	Constitutive expressed UTR46-*blh* gene and *raldh* gene	This study
pSTVM2-blh-Hraldh	Constitutive expressed *blh* gene and UTRH-*raldh* gene	This study
pSTVM2-blh-Mraldh	Constitutive expressed *blh* gene and UTRM-*raldh* gene	This study
pSTVM2-37blh-Hraldh	Constitutive expressed UTR37-*blh* gene and UTRH-*raldh* gene	This study
pSTVM2-37blh-Mraldh	Constitutive expressed UTR37-*blh* gene and UTRM-*raldh* gene	This study
Plasmids for western botting		This study
pET21α(+)	Inducible expression vector, Ap	Novagen
pET21-blh	Induciblyl expressed 6×His-tagged *blh* gene in pET21α(+)	This study
pET21-raldh	Induciblyl expressed 6×His-tagged *raldh* gene in pET21α (+)	This study
Plasmids for genome editing		This study
pRed/ET	Inducible expression Red/ET, Ap	Gene Bridge
pMP11	pKD46 with constitutively expressed Cas9,aTc gRNA targeting ColE1 origin	[13]
pgRNA	Constitutively expressed sgRNA	[13]
pgRNA_malT	Constitutively expressed sgRNA targeting malT	This study

## Data Availability

Not applicable.

## Supplementary Materials

The following are available online at https://www.mdpi.com/article/10.3390/microorganisms9071520/s1. Table S1: Primers used in this study.
