# Supplementary material for: Microbial Production of Bioactive Retinoic Acid Using Metabolically Engineered Escherichia coli
Source: Microorganisms. 2021 Jul 16;9(7):1520. doi: 10.3390/microorganisms9071520 (PMC8305374; doi:10.3390/microorganisms9071520)
Supplement: Supplementary file 1 [file microorganisms-09-01520-s001.zip › microorganisms-1277576-supplementary.pdf]

TableS1. Primers used in this study.

Name	Sequences (5'→3')
**Primers for retinoid biosynthesis pathway construction**	
blh$_{SR}$-F-XbaI	GC**TCTAGA**AGGAGGATTACAAAATGCACAACCCGGTTACCC
blh$_{SR}$-R-EcoRI	G**GAATTC**TCAGGAGACGGCCTGGG
ALDH1A2-F-XbaI	GC**TCTAGA**ATGACTTCCAGCAAGATAGAGAT
ALDH1A2-R-EcoRI	G**GAATTC**TTAGGAGTTCTTCTGGGGGAT
pUCM-F	TCTAGAGCGCCCGGGGA
mRS12-pUCM-R	GTTTAAACTGACTGACGCACCAAAAGCGCTCACAATTCCACACAACA
mRS37-pUCM-R	GTTTAAACAATAAATTACGAGCCAGTCGCTCACAATTCCACACAACA
mRS46-pUCM-R	GTTTAAACCGAATTGGTGGGGCGAGACGCTCACAATTCCACACAACA
mRS-H-pUCM-F-BamHI	CGC**GGATCC**TTTTTGTAGTAAAGGAGCATCGCTAAGGAGGATTACAAATCTAGA ATG
mRS-M-pUCM-F BamHI	CGC**GGATCC**TTTTTGTAGTAAAGAAGCAGGAGGATTACAAATCTAGAATG
mRS-R-BamHI	CGC**GGATCC**CGCTCACAATTCCACACAAC
pET-*blh*$_{SR}$-F-XbaI	GC**TCTAGA**ATGCACAACCCGGTTACCC
pET-*blh*$_{SR}$-R-HindIII	CCC**AAGCTT**GGAGACGGCCTGGGGG
pET-*ALDH1A2*-F-BamHI	CGC**GGATCC**ATGACTTCCAGCAAGATAGAGAT
pET-*ALDH1A2*-R-EcoRI	CG**GAATTC**GGAGTTCTTCTGGGGGATC
pSTVM2-sub-USER-3-F	AGACAGUCATAAGTGCGG
pSTVM2-sub-USER-1-R	ATGCAACUCGTAGGACAG
pUC- sub-USER-3-F	AGACAGUCAATCTGCTCTGATGCC
pUC- sub-USER-1-R	ATGCAACUCATAATGAATCGGCCAAC
pUC-sub-USER-1-F	AGTTGCAUCCCGACTGGAAAGCG
pUC-sub-USER-2-F	ATCCATGUCCCGACTGGAAAGCG
pUC-sub-USER-5-F	ATATGCGAUCCCGACTGGAAAGCG
pUC-sub-USER-2-R	ACATGGAUATGCGGTGTGAAATACC
pUC-sub-USER-5-R	ATCGCATAUATGCGGTGTGAAATACCG
pUC-sub-USER-3-R	ACTGTCUATGCGGTGTGAAATACCG
Gibson-pSTVM2-F	AGACAGTCATAAGTGCGGC
Gibson-*blh*$_{SR}$-F	CCCCCAGAAGAACTCCTAATTCCCGACTGGAAAGCG
Gibson-*blh*$_{SR}$-R	GCCGCACTTATGACTGTCTTATGCGGTGTGAAATACCG
Gibson-*ALDH1A2*-R	TTAGGAGTTCTTCTGGGGG

**Primers for genome editing**	
ybbO-FRT-KO-F	GTTTATTGCCGACTGGATGGCGAAGCAGTTGCAGCCTTTAGTAAATCATGAAATTAACCCTCACTAAAGGGC
ybbO-FRT-KO-R	GACATGGGGGCTTAAGCGCGCGCTTCAACTCACCCCTGCAATATTTTGTCACGACTCACTATAGGGCTC
ybbO-Confirm-F	TCTGCTGAAACAGCATCAG
ybbO-Confirm-R	GTTCTGATTCGACAACTGC
malT-UP-F	AGTCGCTTAAGGCCCTGC
malT-UP-R	TCTTCCAGTGCCAGTTTTGC
malT-Down-F	AGCAAACCGAACAGATGGCA
malT-Down-R	AAGACTTCAATCCCGCTACG
malT-blh_{SR} -F	GCAAAACTGGCACTGGAAGAAGGTTTCCCGACTGGAAAG
malT-blh_{SR} -R	TGCCATCTGTTCGGTTTGCTACTATCGTCGCCGCACTTAT
malT-*ALDH1A2*-F	GCAAAACTGGCACTGGAAGACCCTGTCCTACGAGTTGCAT
malT-*ALDH1A2*-R	TGCCATCTGTTCGGTTTGCTTTAGGAGTTCTTCTGGGGGAT
**Quantitative analysis of gene transcription levels**	
blh_{SR}-RT-F	ATGTCGATGGCCCAGTTC
blh_{SR}-RT-R	GGGTCATGATGGCGATCA
ALDH1A2-RT-F	TATGTGGATTTGCAGGGCGT
ALDH1A2-RT-R	ATTGCCACAGCACAAAGCTG

*Bold letters indicate restriction enzyme sites.

*Underline indicates 5'UTR sequence.
